# In Vitro Evidence on Bioaccessibility of Flavonols and Cinnamoyl Derivatives of Cruciferous Sprouts

**DOI:** 10.3390/nu13114140

**Published:** 2021-11-19

**Authors:** Ángel Abellán, Raúl Domínguez-Perles, Cristina García-Viguera, Diego A. Moreno

**Affiliations:** Phytochemistry and Healthy Foods Lab (LabFAS), Food Science and Technology Department (CEBAS-CSIC), University Campus of Espinardo, 30100 Murcia, Spain; avictorio@cebas.csic.es (Á.A.); rdperles@cebas.csic.es (R.D.-P.); dmoreno@cebas.csic.es (D.A.M.)

**Keywords:** *Brassica*, edible sprouts, hydroxycinnamic acids, flavonols, bioaccessibility, simulated in vitro digestion

## Abstract

Cruciferous sprouts are rising in popularity as a hallmark of healthy diets, partially because of their phytochemical composition, characterized by the presence of flavonols and cinnamates. However, to shed light on their biological activity, the ability to assimilate (poly)phenols from sprouts (bioaccessible fraction) during gastrointestinal digestion needs to be studied. In this frame, the present work studies the effect of the physicochemical and enzymatic characteristics of gastrointestinal digestion on flavonols and cinnamoyl derivatives, by a simulated static in vitro model, on different cruciferous (red radish, red cabbage, broccoli, and white mustard) sprouts. The results indicate that, although the initial concentrations of phenolic acids in red radish (64.25 mg/g fresh weight (fw)) are lower than in the other sprouts studied, their bioaccessibility after digestion is higher (90.40 mg/g fw), followed by red cabbage (72.52 mg/g fw), white mustard (58.72 mg/g fw), and broccoli (35.59 mg/g fw). These results indicate that the bioaccessibility of (poly)phenols is not exclusively associated with the initial concentration in the raw material, but that the physico-chemical properties of the food matrix, the presence of other additional molecules, and the specific characteristics of digestion are relevant factors in their assimilation.

## 1. Introduction

Nowadays, there is increasing consumer concern about the nutritional quality of food, as vegetable origin foods are key for a healthy diet. The role of these plant-based foods in the prevention of a wide array of chronic diseases is associated with their content in bioactive compounds (nutrients and non-nutrients), and to the modification of molecular pathways responsible for the onset and advance of pathophysiological processes [1,2]. Regarding this point, cruciferous sprouts have been demonstrated to be a source of valuable bioactive micronutrients (vitamins and minerals) and phytochemical compounds (glucosinolates and phenolics), related to the prevention of inflammatory, tumoral, or metabolic disorders [3,4].

The value of cruciferous sprouts as dietary sources of bioactive compounds has been demonstrated in recent years. Broccoli (*Brassica olreaceae* var. *italica* L. cv. ‘Calabrese’), red radish (*Raphanus sativus* var. *Sativus* L. cv. ‘Rambo’), white mustard (*Sinapis alba* L.), and red cabbage (*Brassica rapa* L., var. *capitata*) are rich in two major groups of phenolic compounds: glycosylated flavonols and hydroxycinnamic acid derivatives (mainly represented by kaempferol and sinapic acid derivatives) [5,6]. These compounds have been associated with radical scavenging activities at the intracellular level, eliminating reactive oxygen species (ROS) and thus preventing deleterious effects associated with oxidative stress [7].

Phenolic acids are secondary metabolites of higher plants, characterized by a high proportion of glycosylic esters in the raw material. The hydrolytic activity of the digestive enzymes, present in gastric and intestinal mammalian fluids (pepsin, pancreatin, and alkaline phosphatase), break down the glycosylated forms, causing the formation of aglycones and thereby enhancing the concentration of these chemical forms in the intestinal lumen (bioaccessibility) [8]. This is of special relevance because the biological activity of glycosylated flavonols and cinnamic derivatives, present in cruciferous sprouts, is closely dependent on their bioaccessibility [9]. The decrease in the total content of phenolic compounds after the gastric, intestinal and duodenal phases of action of the digestive process of the plant material, has previously been reported [10,11]. However, the different effect of gastrointestinal digestion on the release and stability of phenolic compounds depends on different factors, such as the physicochemical traits of the food matrix, pH, temperature, or enzymatic activity [12,13]. In this regard, a small proportion of the glycosylated forms releases and remains stable under the physicochemical conditions during gastrointestinal digestion, being available to be absorbed in the small intestine, and, in some cases, hydrolyzed towards aglycones (compounds without glycosylated moieties). The formation of these chemical forms alters the polarity and size of these compounds and may modulate their capacity to cross the cell membranes, which contributes to their ability to reach the target cells at an adequate concentration, closely associated with the radical scavenging capacity [2]. In cruciferous foods, these biological properties are strongly associated with sinapic acid derivatives, which have been suggested as responsible for health-promoting bioactivities (anti-inflammatory, anticarcinogenic, and antioxidant effects, among others) [14].

In this scenario, the present work pursues the study of the bioaccessibility of hydroxycinnamic acids and flavonols present in cruciferous sprouts (broccoli, red radish, white mustard, and red cabbage). In addition, it aims to determine the relative contributions of the gastric and intestinal phases of gastrointestinal digestion on the release and stability of cruciferous phenolics, and thereby on the concentration of these bioactive compounds in the digested material and/or their transformation into their bioaccessible aglycones.

## 2. Materials and Methods

### 2.1. Chemicals and Reagents

The standards sinapinic acid and quercetin-3-rutinoside were purchased from Sigma-Aldrich (St Louis, Missouri, USA). Acetic acid, hydrochloric acid, and ammonium acetate were purchased from Panreac (Barcelona, Spain). Methanol, acetonitrile, and acetic acid (LC-MS grade solvents) were provided by J.T. Baker (Philipsburg, NJ, USA). Milli-Q purified water (Millipore, Bedford, MA, USA) was used for all the extraction and chromatographic analyses.

### 2.2. Plant Material

Seeds of broccoli (*Brassica olreacea* var. *italica* L. cv. Calabrese), red radish (*Raphanus sativus* var. *Sativus* L. cv. Rambo), white mustard (*Sinapis alba* L.), and red cabbage (*Brassica rapa* L., var. *capitata*) were provided by Intersemillas S.A. (Loriguilla, Valencia, Spain) certified and untreated. The hygienization and germination of seeds and the preparation of sprouts were performed following established protocols as detailed in Abellan et al. [15].

### 2.3. Processing Brassica Sprout Samples by a Simulated in Vitro Static Digestion Method

Gastric, intestinal, and gastrointestinal digestions were performed on brassica sprout powder (500 mg) following the methodology previously described [16,17] with minor modifications according to Abellan et al. [15]. Simulated gastric and intestinal fluids (SGF and SIF, respectively) were prepared according to the information in Table 1. In brief, pepsin enzyme (EC 3.4.23.1) was prepared by dissolving in SGF at the final concentration of 2000 U/mL and adjusting the pH to 3.0 by adding 1 M HCl. The SGF containing pepsin was added to the samples and continuously stirred at 52 oscillations per min for 2 h at 37 °C in a thermal water bath (Unitronic™ Vaiven, J.P. SELECTA, Barcelona, Spain). During this period, the pH of the SGF was monitored and corrected to ensure a pH of 3.0 during the entire process. The reaction was stopped by adding sodium hydroxide solution (0.2 M), and triplicate samples were analyzed.

After the individual gastric and intestinal digestions developed on the raw material, as well as at the end of the complete gastrointestinal digestion (sequential gastric and intestinal digestions), the samples were centrifuged at 2000 rpm for 5 min at 4 °C in order to separate the soluble or bioaccessible fraction and the residual fraction. The bioaccessible fractions were frozen at −80 °C and freeze-dried. For the extraction of hydroxycinnamic acids and flavonols, the freeze-dried samples were dissolved in 1 mL of MeOH/deionized water (70:30, V/V), sonicated for 30 min, centrifuged at 2000 rpm for 5 min, and filtered through a 0.45 μm filter (Millipore, MA, USA).

### 2.4. HPLC-DAD-ESI/MSn Analysis of Analytical Extracts and Gastrointestinal Digestion Fractions of Brassica Sprouts

**Figure 1:**
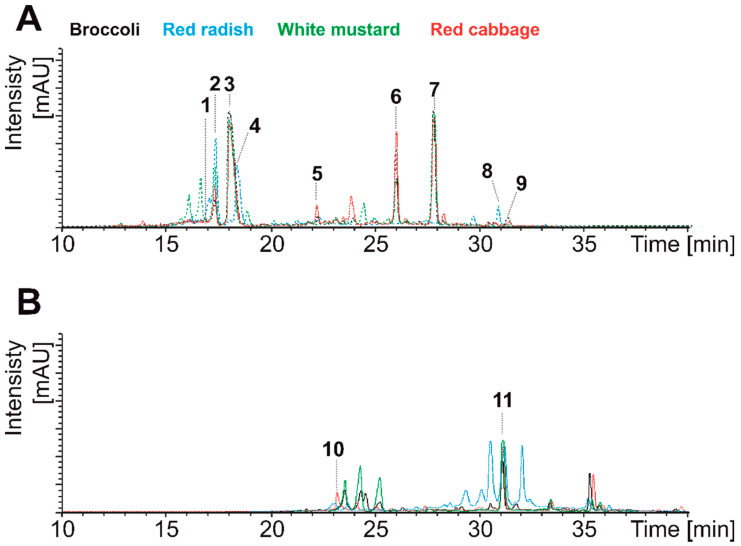
Peak identities in the analytical extracts of plant material (**A**) and the product resulting from the gastrointestinal digestion (**B**): (1) kaempferol sinapoyl-diglcusoside-7-glucoside; (2) sinapoyl-glucose; (3) kaempferol—7-gucoside-3-sinapoyl diglucoside; (4) unknown synapoyl derivative; (5) feruloyl-glucose; (6) disinapoyl-gentibiose; (7) trisinapoyl-gentibiose; (8) disinapoyl-glucose; (9) disinapoyl-glucose (isomer); (10) synapoyl-gentibiose; (11) sinapic acid.

The chromatographic separation of the phenolic compounds, present in the analytical and digestive extracts, was performed on a Kinetex column (5 µm, C18, 100 A, 150 × 4.6 mm) (Phenomenex, Macclesfield, UK). The mobile phases employed consisted of 1% formic acid (A) and acetonitrile (B), starting with 1% B and using a gradient to obtain 25% B at 25 min and 60% B at 40 min. The flow rate was 0.8 mL/min and the injection volume was 20 µL. Spectral data from all peaks were detected in the range 200–600 nm and chromatograms were recorded at 330 nm. The HPLC-DAD-ESI/MSn analyses were carried out in an Agilent HPLC 1100 series equipped with a diode array detector and mass detector in series (Agilent Technologies, Waldbronn, Germany). The HPLC consisted of a binary pump (model G1312A), an autosampler (model G1313A), a degasser (model G1322A), and a photodiode array (PDA) detector (model G1315B). The HPLC system was controlled by ChemStation software (Agilent, v. 08.03). The mass detector was an ion trap spectrometer (model G2445A) equipped with an electrospray ionization interface and was controlled by LCMSD software (v. 4.1, Agilent technologies, Waldbronn, Germany). The ionization conditions were adjusted at 350 °C and 4 kV for capillary temperature and voltage, respectively. The nebulizer pressure and flow rate of nitrogen were 60 psi and 11 L/min, respectively. The full scan mass covered the range *m/z* 100 to 1000. Collision-induced fragmentation experiments were performed in the ion trap using helium as the collision gas, with voltage ramping cycles from 0.3 to 2 V. Mass spectrometry data were acquired in the negative ionization mode. The identification of the hydroxycinnamic acids and flavonols was performed resorting to the retention time (min), parent ions, and fragmentation patterns, in comparison with authentic standards and descriptions available in the literature. Phenolic compounds were characterized and quantified by PDA chromatograms (330 nm), using for each day of analysis freshly prepared calibration curves, with authentic standards (Figure 1 and Table 2).

### 2.5. Statistical Analysis

Results are presented as means ± SD (n = 3). Before selecting the statistical test to be applied for comparison purposes, the normal distribution of the results and the homogeneity of variance were assessed resorting to Kolmogorov–Smirnov and Levene tests, respectively. Because of the normal distribution of the data, an analysis of variance (ANOVA) was applied. When the ANOVA test informed on significant differences, Tukey’s multiple range tests were carried out. The level of statistical significance was set at *p* < 0.01. All statistical analyses were performed using SPSS 25.0 software (LEAD Technologies, Inc., Chicago, IL, USA).

## 3. Results and Discussion

### 3.1. Content of Flavonols and Hydroxycinnamic Acids in the Raw Cruciferous Sprouts

**Figure 2:**
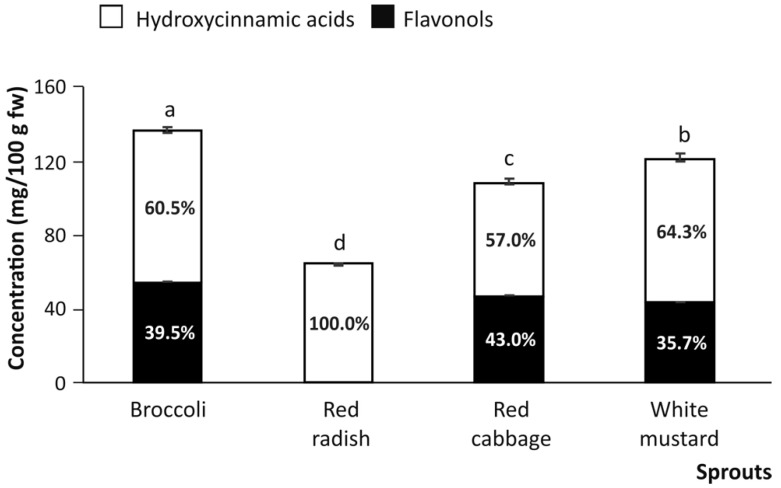
Total content of phenolic compounds (mg/100 g fw) in raw cruciferous sprouts with indication of the percentage of flavonols and hydroxycinnamic acids contributing to the (poly)phenolic profile. Mean values ± SD (n = 3) with different lowercase letters are significantly different at *p* < 0.01 according to the analysis of variance (ANOVA) and Tukey’s multiple range test.

All the cruciferous varieties analyzed in the present work presented a remarkable content of phenolic compounds, resulting from the combination of hydroxycinnamic acids (mainly represented by sinapic acid derivatives) and flavonols (mainly represented by kaempferol derivatives). However, the studied sprouts exhibited significantly different concentrations of total (poly)phenols, as follows: broccoli (137.88 mg/g fresh weight (fw)) > white mustard (120.95 mg/g fw) > red cabbage (108.03 ng/g fw) > red radish (64.25 mg/g fw) (Figure 2). These concentrations are in good agreement with previous descriptions in the literature [18,19,20]. However, in order to understand the biological interest of cruciferous sprouts’ phenolics, the analysis of bioaccessibility needs to be addressed. This will give scientific support for future dietary intake recommendations, effective doses, and nutritional guidelines for human health.

When analyzing the contribution of the phenolic acid derivatives and flavonols to the total (poly)phenolic content, it was found that in broccoli, red cabbage, and white mustard, 60.6%, on average, of phenolic content was represented by hydroxycinnamic derivatives, whereas the remaining 39.4%, on average, corresponded to flavonols. However, the analysis of red radish sprouts evidenced that 100.0% of phenolics were sinapic and ferulic acid glycosides. This information suggested that the limited diversity of phenolic compounds found in red radish sprouts could be responsible for the lower concentration of total phenolics in relation to the other studied sprouts (broccoli, red cabbage, and white mustard) (Figure 2).

Regarding flavonoids, two different flavonols were identified and quantified, with kaempferol-7-glucoside-3-sinapoyl diglucoside being the most abundant, with concentrations ranging between 43.42 and 52.72 mg/g fw in broccoli, red cabbage, and white mustard, while kaempferol sinapoyl-diglucoside-7-glucoside was only detected in broccoli sprouts (Table 2).

However, hydroxycinnamic acid derivatives were mainly represented by sinapic acid derivatives. In this aspect, trisinapoyl-gentibioside was the most abundant hydroxycinnamic acid in white mustard (32.31 mg/g fw), broccoli, and red cabbage sprouts (29.19 mg/g fw, on average). However, sinapoyl-glucose was the most abundant in red radish and white mustard sprouts (22.08 and 36.58 mg/g fw, respectively). Besides, feruloyl-glucose was found mainly in broccoli (18.60 mg/g fw) and, to a lower extent, in red radish sprouts (5.60 mg/g fw) (Table 3).

These concentrations fit well with previous descriptions in the literature on the quantitative phenolic profile of cruciferous sprouts. In this sense, Li et al. did not detect kaempferol or sinapic aglycone in any of the 12 cruciferous sprouts analyzed, but reported a great concentration of glycosylated derivatives as di and tri synapoyl gentibiosides [21]. From a dietary perspective, the composition here described is of special interest due to the closer relationship established between kaempferol derivatives and the prevention of the malignization of cells and, thus, the onset and progressing of tumoral processes [22]. Besides, the presence of hydroxycinnamic acid derivatives here described has been associated with relevant biological effects, such as a significant radical-scavenging power, anticancer activity, and/or antidiabetic effects [23,24]. However, despite the high concentrations found in the studied plant material, the potential biological attributions of the ingested bioactive compounds should be demonstrated. To help to understand this crucial point, the effect of gastrointestinal digestion on the phenolics’ release from the food matrix and their stability remains essential. In this aspect, these studies would provide accurate information on the final bioaccessible fraction available to be absorbed at the intestinal level. Finally, this would exert the biological functions in the target tissues, and would allow establishing rational conclusions regarding the contribution of cruciferous sprouts to human health [19].

### 3.2. Influence of Gastric, Intestinal, and Gastrointestinal Digestion

#### 3.2.1. Gastrointestinal Digestion

The high glycosylation of the phenolic compounds present in the cruciferous sprouts of the present work strongly limits their absorption at the intestinal level and, thereby, the development of their biological functions. However, the diversity of phenolic compounds and glycosylation degree depends on different factors: (1) the dissimilarities detected in the extraction of hydroxycinnamic acids and flavonols during the gastrointestinal digestion, (2) the specific physicochemical conditions in the gastrointestinal tract, and (3) the capacity of the digestive enzymes to break down the ester bonds present in both hydroxycinnamic acids and flavonols. Even more, the sinapic acid aglycones show higher resistance against the physicochemical conditions and enzymatic activity enclosed in the digestion process compared to their glycosylated forms. These characteristics would lead to a greater bioaccessible phenolic fraction due to the high content of sinapic acid aglycones [25,26,27].

**Figure 3:**
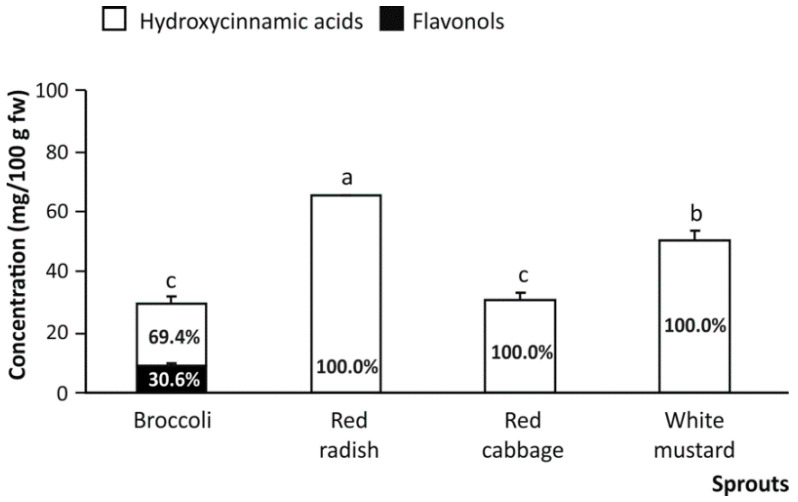
The total content of phenolic compounds (mg/100 g fw) in the products of the gastrointestinal digestion of cruciferous sprouts with indication of the percentage of flavonols and hydroxycinnamic acids contributing to the (poly)phenolic composition. Mean values ± SD (n = 3) with different lowercase letters are significantly different at *p* < 0.01 according to the analysis of variance (ANOVA) and Tukey’s multiple range test.

Besides, continuous gastric and intestinal digestion is efficient to extract the compounds of interest from the food matrix to the intestinal lumen, turning them into available forms, which would be absorbed. Simultaneously, during gastrointestinal digestion the broken-down (poly)phenols are released, to some extent, due to the digestive environment and the enzymatic effects [28,29,30]. Under these conditions, the raw material glycosides would give rise to their respective sinapic and kaempferol aglycones, featured by chemical properties that enhance their intestinal absorption [31]. These mechanisms would be responsible for the decrease in the amount of total phenolic compounds in the products of the gastrointestinal digestion of cruciferous sprouts. In some detail, in digested broccoli, red cabbage, and white mustard sprouts, this reduction was of almost 70%, on average, in comparison with the respective raw materials (Figure 3). In contrast, a better stabilization of hydroxycinnamic acids was observed in the digestion products of red radish, which remained almost unchanged compared to the initial concentration (Figure 3). These results revealed that most degradation of phenolic compounds, present in the cruciferous sprouts, corresponded to the flavonol fraction, which was almost erased during the digestion (with the exception of the digestion products of broccoli sprouts, which retained around 30% of the original flavonol concentration).

Digested plan material quantitative (poly)phenolic results evidenced the formation of the sinapic acid aglycone, which was absent in the intact sprouts. This reaction was observed in all sprouts, being of special relevance in red radish in comparison with the others (Table 4). In addition, other sinapic acid glycosylated derivatives were detected, namely synapoyl-glucose (the major compound present in white mustard), disinapoyl-glucose, and di and tri-sinapoyl-gentibiose (Table 4). Besides, concerning hydroxycinnamic acids, ferulic acid was the major compound detected, presenting losses of 46% during gastrointestinal digestion. In any case, it has been reported that sinapic acid has a higher oxidative power compared to ferulic acid [32].

#### 3.2.2. Gastric and Intestinal Isolate Digestion

**Figure 4:**
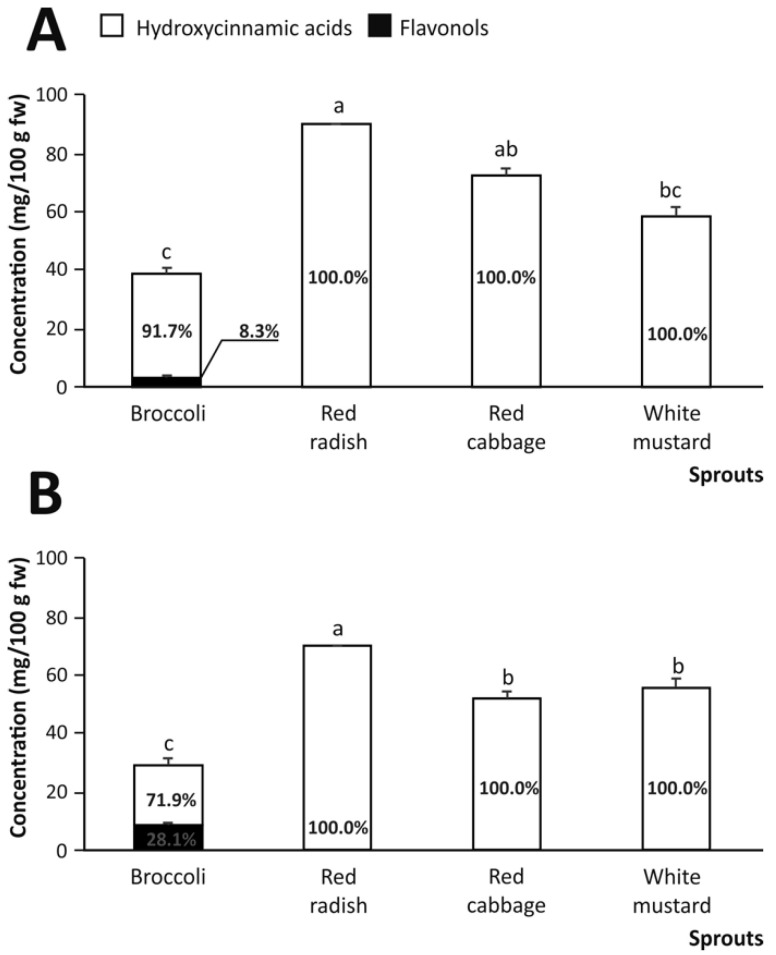
The total content of phenolic compounds (mg/100 g fw) in the fractions of the gastric (**A**) and intestinal (**B**) digestion processes of cruciferous sprouts with the indication of the percentage of flavonols and hydroxycinnamic acids contributing to the (poly)phenolic composition. Mean values ± SD (n = 3) with different lowercase letters are significantly different at *p* < 0.01 according to the analysis of variance (ANOVA) and Tukey’s multiple range test.

To understand the contribution of the two phases of gastrointestinal digestion, the breakdown derivatives obtained from the gastric and intestinal digestion of the (poly)phenols present in the raw material were also analyzed (Figure 3). The results showed an augmented release of phenolic acids into the chyme, produced as a result of the gastric digestion (acid pH and gastric pepsin activity) for all varieties of sprouts (Figure 4A). The decrease in the flavonol content during gastric digestion, detecting only low levels in broccoli sprouts (3.21 mg/100 g fw), was similar to that reported during gastrointestinal digestion. However, red radish sprouts showed the greatest bioaccessibility in comparison with the rest of the sprouts. Finally, a significant difference in total phenolic content was detected in red cabbage sprouts, increasing by 42.26% (72.52 mg/100 g fw) in relation to the gastrointestinal phase (Figure 4A).

However, when applying the physicochemical and enzymatic conditions corresponding to the intestinal digestion to the raw plant material, the bioaccessibility results did not differ significantly from those provided by the gastrointestinal digestion. Nevertheless, considering individually the intestinal digestion of red cabbage sprouts, it can be observed that the bioaccesibility increased by 59.48% (51.70 mg/100 g fw) compared to gastric digestion (Figure 4B).

All the previous results are in agreement with Laib et al., who reported a progressive decrease in the phenolic content of *Camellia sinensis* L. in each digestive stage, from oral phase (mastication) to intestinal phase [33].

The differences observed regarding the efficiency of gastric or intestinal digestion conditions could be explained by analyzing the quantitative profile of the separate digested plant material. In this sense, in a similar way to what was observed during the gastrointestinal phase, the accumulation of sinapic acid aglycone constitutes a key factor for the estimation of the total bioaccessibility of these phenolics, present in the different cruciferous species (Table 5).

Thus, the increase in sinapic acid aglycone during the gastric phase, compared to the intestinal phase, could be a consequence of acid conditions that could stabilize the phenolic compounds, lowering the hydrolysis produced by the gastric and intestinal digestive enzymes [34].

## 4. Conclusions

This study contributes to the understanding of the different effects that gastrointestinal digestion has on the flavonols and hydroxycinnamic acids in cruciferous sprouts, showing the relative contributions of the gastric and intestinal phases. In this sense, it seems that gastric digestion prepares the food matrix for more efficient (poly)phenol extraction during intestinal digestion, the phase in which the highest release and stability of these compounds takes place. Moreover, hydroxycinnamic acids reach higher concentrations than flavonols, making them tentatively more available to be absorbed at the intestinal level.

In addition, despite the high content of phenolic acids present in broccoli, red cabbage, and white mustard sprouts, the red radish sprouts exhibited the greatest bioaccessibility. Finally, the overall results evidence the value of cruciferous sprouts as a dietary source of highly bioaccessible (poly)phenols. However, more work is necessary to understand the capacity of phenolic compounds to cross the intestinal barrier and reach operative concentrations in target tissues and cells in order to develop their biological power.

## Figures and Tables

**Table 1:** Composition of simulated gastric and intestinal fluids (SGF and SIF, respectively).

Consituent	Concentration of SGF, pH 3 (mmol/L)	Concentration of SIF, pH 7 (mmol/L)
Potassium chloride (KCl)	6.90	6.80
Potassium dihydrogenphosphate (KH_2_PO_4_)	0.90	0.80
Sodium hydrogen carbonate (NaHCO_3_)	25.00	85.00
Sodium chloride (NaCl)	47.20	38.40
Magnesium chloride (MgCl_2_)	0.10	0.33
Ammonium carbonate ((NH_4_)CO_3_)	0.50	-

**Table 2:** Qualitative HPLC-DAD-ESI/MSn analysis of the individual hydroxycinnamic acids and flavonols present in raw material and digested broccoli (*Brassica olreacea* var. *italica* L. cv. ‘Calabrese’), red radish (*Raphanus sativus* var. *Sativus* L. cv. ‘Rambo’), white mustard (*Sinapis alba*), and red cabbage (*Brassica rapa,* var. *capitata*) extracts.

Compound	Parent Ion (*m/z* [M-H]^-^)	Product Ions (*m/z* [M-H]^-^)		Broccoli Sprouts				Red Radish Sprouts				Red Cabbage Sprouts				White Mustard Sprouts			
		(MS2)	(MS3)	PM	GP	IP	GIP	PM	GP	IP	GIP	PM	GP	IP	GIP	PM	GP	IP	GIP
Kaempferol sinapoyl-diglcusoside-7-glucoside	977	815,609,429	285	X	X	X	X	-	-	-	-	-	-	-	-	-	-	-	-
Sinapoyl-glucose	385	265,247	223	X	-	X	X	X	-	X	X	X	-	X	X	-	-	-	-
Kaempferol—7-gucoside-3-sinapoyl diglucoside	977	623,429	285	X	-	X	X	-	-	-	-	X	-	-	-	X	-	-	-
Unknown synapoyl derivative	236	207	223	-	-	-	-	-	X	-	-	-	X	-	-	-	X	-	-
Feruloyl-glucose	385	355,244	193	X	-	X	X	X	-	-	-	-	-	-	-	-	-	-	-
Disinapoyl-gentibioside	753	529,289	223	X	-	X	X	-	-	X	X	X	-	-	X	X	-	X	X
Trisinapoyl-gentibioside	959	753,529,289	223	X	-	X	X	X	-	X	X	X	-	X	X	X	-	X	X
Disinapoyl-glucose	591	367,223,193	223	-	-	-	-	X	-	-	-	-	-	-	-	X	-	-	-
Disinapoyl-glucose (isomer)	591	367,193	223	X	-	-	-	-	-	-	-	-	-	-	-	-	-	-	-
Synapoyl-gentibioside	547	529,247	223	-	-	-	-	-	-	-	-	-	-	X	X	X	-	X	X
Sinapic acid	223	207,179	163	-	-	-	-	-	X	X	X	X	X	X	X	-	X	X	X

PM, plant material; GP, gastric phase; IP, intestinal phase; GIP, gastrointestinal phase. Compounds corresponding with the peaks shown in Figure 1.

**Table 3:** Quantitative profile (mg/100 g fw) of phenolic compounds of broccoli (*Brassica olreacea* var. *italica* L. cv. ‘Calabrese’), red radish (*Raphanus sativus* var. *Sativus* L. cv. ‘Rambo’), white mustard (*Sinapis alba* L.), and red cabbage (*Brassica rapa* L. var. *capitata*).

Compound	Sprouts			
	Broccoli	Red Radish	Red Cabbage	White Mustard
Flavonols				
Kaempferol sinapoyl-diglucoside-7-glucoside	1.18 ± 0.01 a ^Z^	N.d. ^Y^ b	N.d. b	N.d. b
Kaempferol—7-glucoside-3-sinapoyl diglucoside	52.72 ± 0.77 a	N.d. d	46.44 ± 0.57 b	43.42 ± 0.06 c
Hydroxycinamic acids				
Sinapoyl-glucose	5.89 ± 0.05 d	22.08 ± 0.70 b	9.99 ± 0.08 c	36.58 ± 1.41 a
Unknown sinapoyl derivative	N.d. b	21.31 ± 0.05 a	N.d. b	N.d. b
Feruloyl-glucose	18,60 ± 0.87 a	5.60 ± 0.14 b	N.d. c	N.d. c
Disinapoyl-glucose	17.30 ± 1.50 a	11.70 ± 1.06 b	N.d. c	N.d. c
Disinapoyl-gentibioside	13.79 ± 0.36 b	N.d. d	21.72 ± 2.93 a	8.85 ± 0.13 c
Trisinapoyl-gentibioside	28.40 ± 1.05 a	3.55 ± 0.30 b	29.89 ± 2.93 a	32.31 ± 1.87 a

^Z^ Data presented as a mean ± SD (n = 3). Values followed by different lowercase letter within the same row are significantly different at *p* < 0.01 according to the analysis of variance (ANOVA) and Tukey’s multiple range test. ^Y^ N.d., not detected.

**Table 4:** Quantitative profile (mg/100 g fw) of individual flavonols and hydroxycinnamic acids of the products of the gastrointestinal digestion of cruciferous sprouts.

Compound	Sprouts			
	Broccoli	Red Radish	Red Cabbage	White Mustard
Flavonols				
Kaempferol sinapoyl-diglucoside-7-glucoside	3.55 ± 0.27 a ^Z^	N.d. b	N.d. b	N.d. b
Kaempferol—7-gucoside-3-sinapoyl diglucoside	4.59 ± 0.11 a	N.d. b	N.d. b	N.d. b
Hydroxycinnamic acids				
Sinapoyl-glucose	2.94 ± 0.39 b	2.02 ± 0.24 b	3.61 ± 0.36 b	29.00 ± 3.69 a
Synapoyl-gentibioside	N.d. b	N.d. b	3.82 ± 0.34 a	N.d. b
Unknown synapoyl derivative	N.d. b	2.09 ± 0.18 a	N.d. b	N.d. b
Feruloyl-glucose	8.61 ± 0.20 a	N.d. b	N.d. b	N.d. b
Sinapic acid	1.40 ± 0.05 c	45,39 ± 2.65 a	15.54 ± 2.48 ab	17.93 ± 1.61 ab
Disinapoyl-glucose	N.d. b	N.d. b	N.d. b	1.90 ± 0.25 a
Disinapoyl-gentibioside	1.77 ± 0.17 b	14.71 ± 2.36 a	N.d. c	1.62 ± 0.22 b
Trisinapoyl-gentibioside	6.08 ± 0.29 b	1.10 ± 0.17 c	7.67 ± 0.28 a	N.d. d

^Z^ Data presented as a mean ± SD (n = 3). Values followed by different lowercase letters within a row are significantly different at *p* < 0.01 according to the analysis of variance (ANOVA) and Tukey’s multiple range test.

**Table 5:** Quantitative profile (mg/100 g fw) of individual flavonols and hydroxycinnamic acids of the gastric and intestinal digested plant material.

Compound	Digestive Phase ^Z^	Sprouts			
		Broccoli	Red Radish	Red Cabbage	White Mustard
Flavonols					
Kaempferol sinapoyl-diglucoside-7-glucoside	GD	3.21 ± 0.30 b ^Y^	N.d. ^X^ b	N.d. b	N.d. b
	ID	4.55 ± 0.22 a	N.d. b	N.d. b	N.d. b
Kaempferol—7-glucoside-3-sinapoyl diglucoside	GD	N.d. b	N.d.	N.d.	N.d.
	ID	4.32 ± 0.07 a	N.d. b	N.d. b	N.d. b
Hydroxycinammic acids					
Sinapoyl-glucose	GD	N.d.	N.d.	N.d.	N.d.
	ID	2.69 ± 0.02 b	1.83 ± 0.28 b	4.59 ± 0.06 b	27.11 ± 1.52 a
Synapoyl-gentibioside	GD	N.d.	N.d.	N.d.	N.d.
	ID	N.d. b	N.d. b	5.03 ± 0.69 a	N.d. b
Unknown synapoyl derivative	GD	N.d.	N.d. c	N.d.	N.d.
	ID	N.d. b	2.97 ± 0.20 a	N.d. b	N.d. b
Feruloyl-glucose	GD	N.d.	N.d.	N.d.	N.d.
	ID	10.19 ± 0.38 a	N.d. b	N.d. b	N.d. b
Sinapic acid	GD	35.59 ± 0.49 b	58.32 ± 7.89 a	45.38 ± 4.06 ab	32,08 ± 0.32 b
	ID	0.63 ± 0.09 c	47.34 ± 5.11 a	26.29 ± 0.56 b	25.45 ± 0.45 b
Disinapoyl-glucose	GD	N.d.	N.d.	N.d.	N.d.
	ID	N.d. b	N.d. b	N.d. b	1.51 ± 0.12 a
Disinapoyl-gentibioside	GD	N.d.	N.d.	N.d.	N.d.
	ID	2.69 ± 0.10 c	16.26 ± 1.61 a	4.31 ± 0.39 b	1.39 ± 0.15 c
Trisinapoyl-gentibioside	GD	N.d.	N.d.	N.d.	N.d.
	ID	4.49 ± 0.12 b	1.32 ± 0.13 c	11.47 ± 0.91 a	N.d. d
Unknown synapoyl derivative	GD	N.d. c	32.08 ± 4.11 a	27.15 ± 3.14 a	22,38 ± 1.93 b
	ID	N.d.	N.d.	N.d.	N.d.

^Z^ GD, gastric digestion products; ID, intestinal digestion products. ^Y^ Data presented as a mean ± SD (n = 3). Values followed by different lowercase letters within a column represent significant differences between sprouts at *p* < 0.01 according to the analysis of variance (ANOVA) and Tukey’s multiple range test. ^X^ N.d., not detected.
